# Learning hierarchical sequence representations across human cortex and hippocampus

**DOI:** 10.1126/sciadv.abc4530

**Published:** 2021-02-19

**Authors:** Simon Henin, Nicholas B. Turk-Browne, Daniel Friedman, Anli Liu, Patricia Dugan, Adeen Flinker, Werner Doyle, Orrin Devinsky, Lucia Melloni

**Affiliations:** 1 New York University Comprehensive Epilepsy Center, 223 34th Street, New York, NY 10016, USA.; 2 Department of Neurology, New York University School of Medicine, 240 East 38th Street, 20th Floor, New York, NY 10016, USA.; 3 Department of Psychology, Yale University, New Haven, CT, USA.; 4 Department of Neuroscience, Max Planck Institute for Empirical Aesthetics, Grüneburgweg 14, 60322 Frankfurt am Main, Germany.

## Abstract

Sensory input arrives in continuous sequences that humans experience as segmented units, e.g., words and events. The brain’s ability to discover regularities is called statistical learning. Structure can be represented at multiple levels, including transitional probabilities, ordinal position, and identity of units. To investigate sequence encoding in cortex and hippocampus, we recorded from intracranial electrodes in human subjects as they were exposed to auditory and visual sequences containing temporal regularities. We find neural tracking of regularities within minutes, with characteristic profiles across brain areas. Early processing tracked lower-level features (e.g., syllables) and learned units (e.g., words), while later processing tracked only learned units. Learning rapidly shaped neural representations, with a gradient of complexity from early brain areas encoding transitional probability, to associative regions and hippocampus encoding ordinal position and identity of units. These findings indicate the existence of multiple, parallel computational systems for sequence learning across hierarchically organized cortico-hippocampal circuits.

## Teaser

Sequence learning is tracked across the human brain, and the computational systems used to parse the sequences are highlighted.

## INTRODUCTION

We receive continuous input from the world and yet experience it in digestible chunks. In the domain of language, for example, acquisition and use require extracting meaningful sequences such as words, phrases, and sentences out of a continuous stream of sounds, often without clear acoustic boundaries or pauses between linguistic elements (*1*). This segmentation ability occurs incidentally and effortlessly and is thought to be a core building block of development. Young infants can learn transitional probabilities (TPs) between syllables (*2*) or shapes (*3*) to extract embedded regularities after minimal exposure. In a seminal study (*2*), 8-month-old infants segmented words after brief exposure to a continuous sequence of an artificial language in which TPs between syllables indicated word boundaries. Since this discovery, similar abilities have been demonstrated in adults (*4*, *5*), who also rely on TPs and other statistical properties (*6*–*8*). This behavior—referred to as “statistical learning” (SL)—occurs across many different sensory modalities, tasks, and even species. SL represents a fundamental behavior, and yet the brain mechanisms that support this cognitive function are poorly understood.

Brain regions such as the hippocampus and the inferior frontal gyrus (IFG) have been implicated in visual (*9*, *10*) and auditory SL (*10*, *11*). As previous studies have focused on how the brain changes after SL, the role of these brain areas during the acquisition of statistical regularities remains largely unexplored. Even less is known about what information is represented in these learned regularities and whether sequences are encoded similarly or in a complementary fashion across these brain areas. Regularities extracted during SL range from simple to complex, including TPs between adjacent elements (i.e., uncertainty given a local context), ordinal position in a sequence (i.e., whether an element takes the first, second, third, etc. position), and the identity of the learned unit (i.e., a specific higher-order chunk such as a word) (*12*). Last, the fact that SL has been observed across sensory modalities raises the question of whether the same brain areas and algorithms support extraction and representation of regularities (*13*).

To answer these questions, we collected intracranial recordings [electrocorticography (ECoG)] from 23 human epilepsy patients with broad cortical and hippocampal coverage during an SL task. We used neural frequency tagging (NFT) (*14*, *15*) to identify recording sites responsive to the underlying regularities of the SL stimuli over different time scales (e.g., syllables and words). NFT leverages the fact that cortical activity tracks the rhythms of hierarchical linguistic structure, thus allowing the “tagging” of frequency-specific linguistic activity. Combining ECoG and NFT, we describe the location and temporal tuning of the neural response. Following identification of responsive sites, we used representational similarity analysis (RSA) to determine which aspect(s) of the temporal regularities are represented, i.e., TPs, ordinal position, and identity. Last, we relate the neural circuits, online dynamics, and representational changes for SL across auditory and visual modalities.

We found that SL occurs quickly in both auditory and visual modalities. In both modalities, partially overlapping neural circuits encoded statistical units (e.g., words and fractal pairs) and their constituent sensory elements (e.g., syllables and images). This learning was supported by rapid changes in the similarity space of neural representations, with structure encoded at multiple levels: (i) TPs, with elements grouped by probability strength; (ii) ordinal position, with elements grouped by sequence order; and (iii) identity, with elements grouped by unit in which they are embedded. Auditory and visual elements underwent similar SL-related representational changes, yet involved brain areas only partially overlapped [generally supramodal areas, such as IFG, anterior temporal lobe (ATL), and the hippocampus]. These results provide mechanistic insight into a fundamental human learning ability, revealing how cortical areas respond to the structure of the world. Our findings also highlight NFT as a versatile tool for investigating incidental learning in preverbal infants and other nonverbal patient populations.

## RESULTS

### Behavioral evidence of auditory SL

**Fig. 1:**
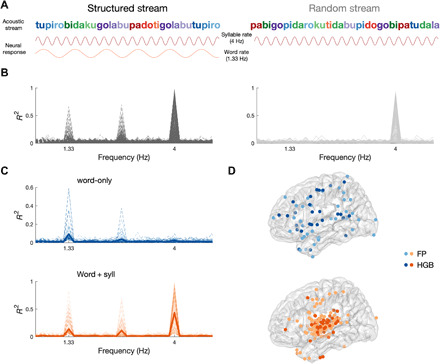
Neural tracking of auditory SL. (**A**) Schematic depiction of the auditory SL task. The structured stream (left) contained 12 syllables [250-ms stimuli onset asynchrony (SOA), 4 Hz] in which the TPs formed four words (color-coded for visualization, 750-ms SOA, 1.33 Hz). The random stream (right) contained the same 12 syllables in a random order. The predicted neural response is shown below each syllable stream: Syllable tracking (top) was expected in both conditions, whereas word tracking (bottom) was expected only in the structured condition. (**B**) Phase coherence spectrum in neural data for the structured (left, black) and random (right, gray) conditions from 1898 electrodes in 17 patients. Each significant electrode is depicted with a thin line, and the average is depicted with a thick line. (**C**) Phase coherence spectrum in the structured condition for electrodes showing word-tracking responses, in two groups: electrodes that showed tracking responses at the word rate only (top, blue) and electrodes that showed tracking responses at both the word and syllable rate (bottom, orange). (**D**) Localization of word-only (top, blue) and word + syll (bottom, orange) electrodes exhibiting significant phase coherence in the field potential (FP; light blue, light orange) or the high-gamma band (HGB; dark blue, dark orange).

To investigate the neural circuits and computations underlying SL, we presented a group of 17 epilepsy patients with brief (2 min × 5 blocks) auditory streams of syllables in which the structure of the sequence was manipulated. In structured streams, each syllable was placed into the first, second, or third position of a three-syllable word or “triplet” (Fig. 1A). A continuous stream of syllables was generated by randomly inserting multiple repetitions of each word without pauses or prosodic cues between words. In random streams, syllables were inserted the same number of times but in a random order at the syllable level. Thus, the TPs were low and uniform, without a word-level of segmentation.

Participants were not informed about the stimulus structure. They were asked to perform a one-back cover task, in which they had to detect occasional repetitions of individual syllables that had been inserted into both stream types (*16*). This task has been used to evaluate SL online while assuring attention to the SL stimulus. Accuracy in both streams was high and not statistically different [*t*(16) = 2.03, *P* = 0.06], indicating that participants attended to the stimuli across both the structured [mean *d′* = 1.04, *t*(16) =14.41, *P* < 0.01] and random [mean *d′* = 0.87, *t*(16) = 14.27, *P* < 0.01] streams. Critically, we found that behaviorally, reaction times to repeated syllables in the structured stream (mean = 733 ms) were significantly faster than in the random stream (mean = 917 ms; *Z* = −3.3, *P* = 0.001, *N* = 17; fig. S2), suggesting that facilitation had occurred because of learning of the underlying structure.

Immediately after exposure to both streams, participants were informed of the hidden structure and were asked to perform an explicit recognition task. Recognition of the hidden words in the structured stream was assessed using a two-alternative forced choice (2AFC) task between the hidden words and part-words. Part-words consisted of previously shown sequences of syllables but that spanned words and thus had overall lower TPs. Across subjects, offline explicit recognition of the hidden words did not exceed chance (50%) performance (mean = 45.4%, SD = 10%; *Z* = −1.84, *P* = 0.07, *N* = 15).

The same procedure was used in a separate cohort of healthy subjects in which we replicated the online incidental learning effect in the reaction times, i.e., faster responses to syllable repetition in the structured than in then random condition (mean structured = 625 ms, mean random = 828 ms, *Z* = −3.8, *P* < 0.001, *N* = 18). Offline explicit recognition was significantly better than chance in this neurotypical cohort (mean = 57.3%, *Z* = 2.04, *P* = 0.04, *N* = 18; fig. S2).

### Neural tracking of auditory SL

We obtained direct neurophysiological signals from 1898 intracranial electrodes in the 17 participants, comprehensively covering the frontal, parietal, occipital, and temporal lobes and the hippocampus in both hemispheres (fig. S1). We capitalized on NFT to evaluate the temporal dynamics of the neural activity to scout for cortical areas responding at the rate of the learned regularities. The sensitivity of NFT to track SL has been previously demonstrated using noninvasive techniques, i.e., electroencephalography (EEG) and magnetoencephalography (MEG) (*17*, *18*), enabling us to definitively resolve the cortical areas exhibiting selective temporal tuning to the learned regularities. Specifically, NFT was used to track representations of segmented units at two hierarchical levels of the stream (*14*, *15*, *17*). We expected entrainment at the syllabic frequency (4 Hz) in both structured and random streams. In contrast, we expected that entrainment at the word-level frequency (corresponding to triplet boundaries or 1.33 Hz) should emerge during exposure to the structured but not random stream, consistent with segmentation of the structured stream.

We first evaluated within-electrode phase coherence in both the field potential (FP) and the envelope of the high-gamma band (HGB) (*15*) in the structured and random streams, respectively. Consistent with our hypothesis, we found electrodes that showed a significant peak in their phase coherence spectrum at the syllable rate (i.e., 4 Hz) for both structured and random streams [criterion for observing a significant response, false discovery rate (FDR)–corrected *P* < 0.05]. In addition, there was a significant peak at the word rate (i.e., 1.33 Hz), but only for the structured stream (criterion for observing a significant response: *P* < 0.05, FDR-corrected; Fig. 1B). These responses were observed predominately in somatosensory/motor and temporal cortices (fig. S4). In these electrodes, we also observed a significant phase coherence peak at 2.66 Hz for the structured stream, possibly reflecting an oscillation at the rate of syllable pairs, consistent with evidence that participants can learn sequential pairs embedded in triplets, in addition to the triplets themselves (*19*), or, alternatively, a harmonic of the word rate or a beat frequency. The word-rate response in the structured stream emerged rapidly, with a significant response observed as early as 50 word exposures in some electrodes (fig. S5). On average, significance of word-rate coherence increased over time in the structured stream but not in the random stream (fig. S5A), ruling out effects of endogenous entrainment over time unrelated to learning. Coherence at the word rate in the structured stream was replicated across participants, with 16 of 17 patients exhibiting significant entrainment (table S1); by contrast, no electrodes showed entrainment at the word rate for the random stream. This finding further supports NFT as a sensitive and robust tool for assessing online SL.

We then exploited the unique spatial resolution afforded by ECoG to localize which cortical areas became synchronized to the word rate in the structured condition (Fig. 1D). Different temporal tuning responses were observed across electrodes. One tuning profile corresponds to electrodes showing significant coherence at both the word and syllable rate (word + syll). These were located primarily in the superior temporal gyrus (STG), with smaller clusters in motor cortex and pars opercularis. The other tuning profile reflected electrodes showing significant coherence exclusively at the word rate (word-only). These were located in IFG and ATL (Fig. 1D). These functional responses indicate temporal selectivity to both the input (in this case, the syllable) and higher-order learned units (for word + syll electrodes), or to higher-order learned units alone without responding to specific acoustic features of the input conveying the structure (for word-only electrodes). This organization reflects the neuroanatomy of the auditory processing hierarchy, with lower-order function in STG and higher-order function in surrounding fronto- and temporo-parietal cortex (*20*). Thus, we reasoned that word-only responses may arise from higher-level stages of processing than word + syll responses. To quantify this anatomical grouping by electrode type, we tested the hypothesis that electrodes belonging to one type (i.e., word-only or word + syll) tend to group together (e.g., nearest electrode was of the same type) using a Bayesian binomial test. Bayesian analysis provided evidence in support of this hypothesis [nearest electrode in “same type” versus “different type,” log(BF10) = 40.75].

### Representational analysis in auditory SL

The NFT results, so far, provide evidence of segmentation of the continuous auditory stream with characteristic tuning in lower-order areas in STG and higher-order areas in surrounding fronto- and temporoparietal cortex. Having mapped the responsive electrodes through NFT, we then asked the question: what is driving this segmentation? The neural response to segmentation could be based on at least three statistical cues in the stream: TPs (within word, TP = 1.0; between word, TP = 0.33), ordinal position (first, second, or third position), or word identity (blue, green, purple, or red word, as in colors from Fig. 1A). Although all three cues could be used to mark the start and end of the words and thus drive segmentation, they differ in content and facilitate unique cognitive functions. For instance, coding based on TPs and the entailed difference in entropy between high and low TP can serve as a strong prediction error cue to drive attention and segmentation. Coding of ordinal position represents a flexible and abstract code allowing the recombination of elements and might explain previous findings on phantom words (*21*), whereby subjects accept as legal, strings that have never appeared during the exposure phase as long as ordinal position is preserved. Yet, only coding based on identity gives access to whole words, which can then be mapped onto meaning (*22*).

To evaluate what information is being represented, we used a multivariate pattern similarity approach. In the case of word identity, for example, we reasoned that SL would change the representational space of stimuli such that syllables belonging to the same word would evoke more similar neural activity patterns across electrodes (*9*); this clustering could, in turn, provide a basis for segmentation (*7*). Alternatively, the neural representations of syllables may cluster by ordinal position or TP, allowing us to test which of these cues was learned and whether similar or complementary codes are observed across brain areas. We quantified the representational space of syllables within the sets of electrodes identified as exhibiting word-only and word + syll coherence (see Fig. 1C) in the NFT analysis (either FP or HGB). In addition, we separately investigated neural representations across electrodes in the hippocampus, as previous studies have shown that the hippocampus is necessary for robust SL (*10*, *23*). We calculated the correlation distance between the patterns of raw neural activity across electrodes within each set of electrodes, i.e., word-only, word + syll, and hippocampus, separately, for each pair of syllables and applied multidimensional scaling (MDS) to visualize the similarity structure.

**Fig. 2:**
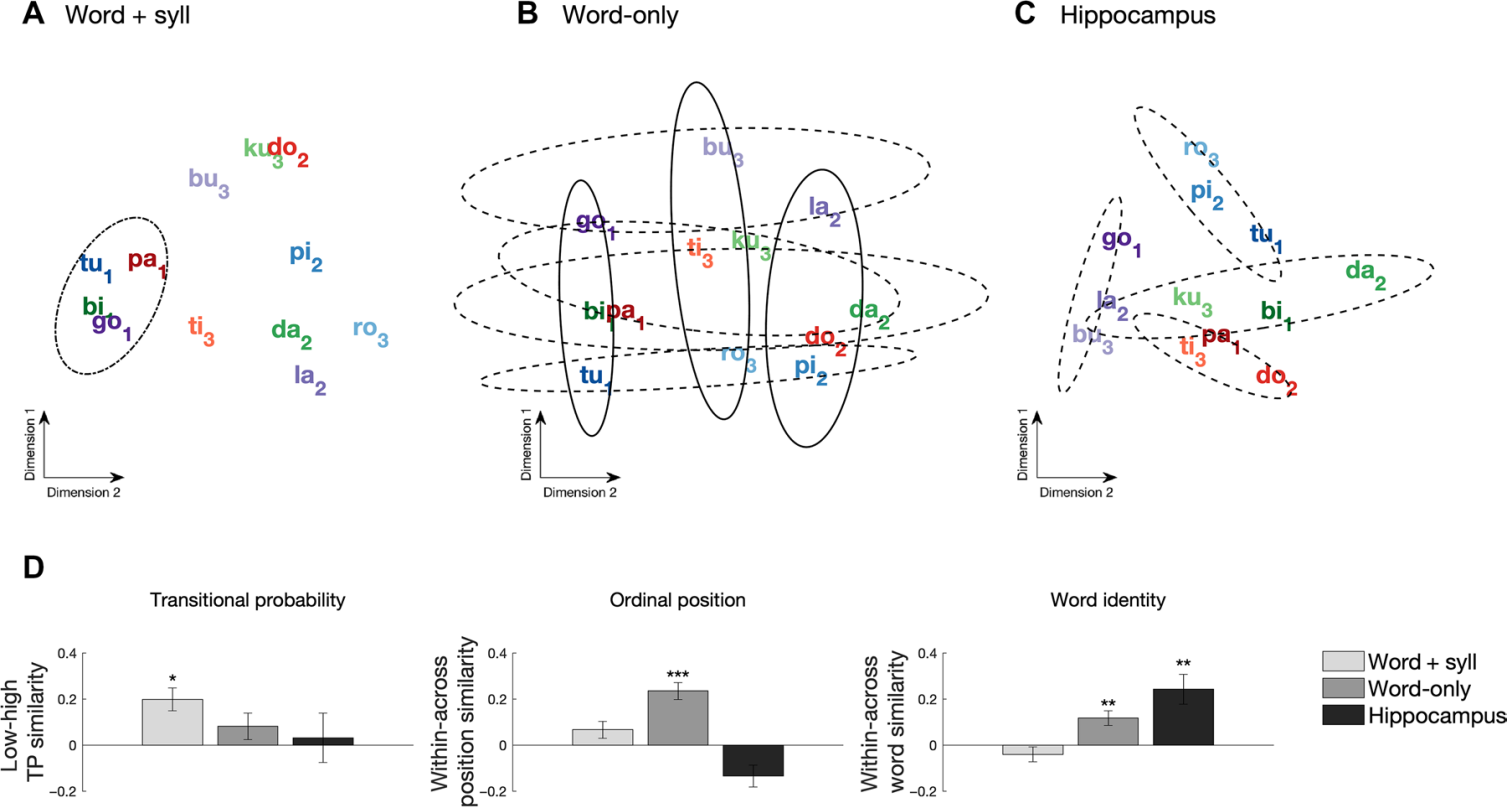
Pattern similarity results during auditory SL. Multidimensional scaling (MDS) of the distances between syllabic responses across electrodes showing significant (**A**) word + syll responses and (**B**) word-only responses, as well as (**C**) across electrodes from the hippocampus. Individual words are color-coded; subscripts represent ordinal position (e.g., “tu_1_pi_2_ro_3_”). Dot-dashed ellipses indicate grouping by TP, solid ellipses outline grouping by ordinal position, and dashed ellipses indicate grouping at the level of the individual words (color-coded). (**D**) Quantification of multivariate similarity for syllables in the auditory SL task. Left: Similarity by TP. Greater within-class similarity indicates stronger grouping of syllables with low TP (0.33) than syllables with high TP (1.0). A Friedman test indicated a main effect of electrode type on TP similarity (χ^2^ = 22.03, *P* < 0.001). Middle: Within versus between similarity for ordinal position. Greater within-class similarity indicates stronger grouping of syllables holding the same first, second, or third position in a word. A Friedman test indicated a significant main effect of electrode type (χ^2^ = 790.35, *P* < 0.001). Right: Within versus between similarity for word identity. Greater within-class similarity indicates grouping of syllables into individual words. A Friedman test indicated a significant main effect of electrode type (χ^2^ = 265.29, *P* < 0.001). ***P < 0.001 and **P < 0.01, Bonferroni-corrected Wilcoxon rank sum test; error bars denote the population SEM.

We found that the three sets of electrodes encoded different information: For word + syll electrodes, distances between syllables revealed a representation based on TPs only (Fig. 2A), grouping syllables based on whether their probability given the preceding syllable was low (first) versus high (second, third). In contrast, word-only electrodes represented ordinal position (Fig. 2B), grouping syllables based on which position they occupied in the words (first versus second versus third) and based on the identity of the word to which they belong (Fig. 2B, dimension 2). Last, hippocampal electrodes showed grouping by word identity only (Fig. 2C). To quantify the apparent grouping effects in each set of electrodes, we used an electrode resampling approach to examine pattern similarity across syllables for groupings consistent with TP, ordinal position, and word identity. To that end, we compared pattern similarity (correlation) for within (same class) versus between (different classes) categories; i.e., similarity of syllables with low versus high TP, same versus different ordinal position, and same versus different word identity, across a set of 200 random resamples for each electrode type (word-only, word + syll, hippocampus). Consistent with the visualized groupings observed in the MDS analysis, low TP coding was only observed for word + syll electrodes (Fig. 2D, left). Reliable coding for ordinal position was observed for the word-only electrodes (Fig. 2D, middle). Word identity was observed both in word-only electrodes and in the hippocampus (Fig. 2D, right). We repeated this analysis using a conceptual model-based dissimilarity comparison (*24*) and observed the same coding schema for each electrode type (fig. S20). These results show that even brief exposure to auditory regularities can reshape the representational space of syllables throughout cortex and the hippocampus, giving rise to clustered neural representations along several dimensions. That is, learning of sequences shapes representations at multiple levels concurrently, with a division of labor across lower- and higher-order brain areas in terms of simple and generic versus complex and specific regularities. The clustering of responses by TP, ordinal position, and identity is consistent with fast learning during exposure, as they were absent during the first block (~2 min) and present by the fifth block (fig. S6). Clustering of responses by these various coding schemes was not observed when the same analysis was performed on the HGB envelope using the same sets of electrodes (fig. S8). Moreover, no clustering of syllable representations was observed in any electrode set when the same analysis was performed on the random stream (fig. S9). This demonstrates that changes in representational space for the structured stream resulted from SL and not properties of the individual stimuli per se. We further analyzed electrode types by field type (e.g., FP-responsive versus HGB-responsive electrodes) and found that these general coding strategies were preserved by response type (word + syll, word-only), with the exception that only HGB-responsive electrodes appeared to provide evidence for coding by word identity (fig. S7).

### Behavioral evidence of visual SL

**Fig. 3:**
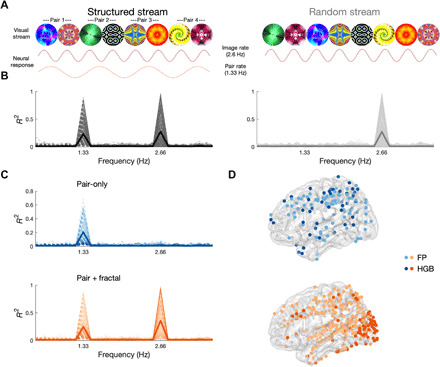
Neural tracking of visual SL. (**A**) Schematic depiction of the visual SL task. The structured stream (left) consisted of a continuous visual stream of eight fractals (375-ms SOA, 2.66 Hz). The TPs were adjusted to form four fractal pairs (750-ms SOA, 1.33 Hz). Note that the SOA of the fractals was elongated compared to the syllables to match the frequency of the learned units (pairs and words), given that there were two fractals per unit and three syllables. The random stream (right) contained the same fractals but in random order. The predicted neural responses are shown under each stream: Fractal tracking is expected for both streams, while pair tracking is expected for the structured stream only. (**B**) Phase coherence spectrum in neural data for the structured (left, black) and random (right, gray) conditions from 1606 electrodes in 12 patients. Each significant electrode is depicted with a thin line, and the average across the population is depicted with a thick line. (**C**) Phase coherence spectrum in the structured condition for electrodes showing pair-tracking responses, in two sets: electrodes that tracked pairs only (left, blue) and electrodes that tracked pairs and fractals (right, orange). (**D**) Localization of pair-only (top, blue) and pair + fractal (bottom, orange) electrodes exhibiting significant phase coherence in the FP (light blue, light orange) or HGB (dark blue, dark orange).

Segmenting continuous input into discrete units extends also to stimuli in the visual domain, e.g., to build representations of scenes and events (*25*). Controversy remains as to whether similar SL mechanisms are engaged in segmenting and acquiring structure across auditory and visual domains (*13*). To investigate whether similar coding principles could be at work in the visual modality, we tested visual SL in 12 intracranial patients. These participants were exposed to brief (2 min × 5 blocks) visual streams of fractal images (375 ms each) in which the structure of the sequence was manipulated (*9*). In the structured streams, each fractal was assigned to the first or second position of a pair (Fig. 3A). We generated a continuous stream of fractal pairs by randomly inserting each pair without breaks or other cues between pairs. To make the results comparable across modalities, we kept the higher-order unit rate, i.e., 1.33 Hz, and thus presented pair of fractals. In the random streams, fractals were inserted the same number of times but in a random order at the fractal level. As a result, there were no pairs to segment.

As in the auditory sequence, participants were not informed about the presence of structure in some of the sequences. Instead, they were asked to perform a one-back cover task, in which they had to detect repetitions of individual fractals that had been occasionally inserted into both stream types (*16*). Accuracy was similarly high in both structured [mean *d*′ = 2.46, *t*(7) = 10.27, *P* < 0.001] and random [mean *d*′ = 2.29, *t*(7) = 11.6, *P* < 0.001] streams and did not differ [*t*(7) = 0.89, *P* = 0.40]. This suggests that participants were equally engaged and attentive during both streams. Consistent with incidental SL of the structure, we again found significantly faster reaction times in the structured stream (mean = 609 ms) than in the random stream (mean = 705 ms; *Z* = −2.24, *P* = 0.03, *N* = 8; fig. S10).

Following exposure to both streams, participants performed a 2AFC recognition task to assess explicit learning of the fractal pairs. Average offline explicit recognition was at chance performance in these participants (mean = 53.3%, SD = 8%; *Z* = 1.23, *P* = 0.22, *N* = 12). However, as with auditory SL, we again replicated the findings in a neurotypical sample and found evidence of incidental learning in the reaction times (e.g., faster reaction times in the structured condition; mean structured = 630 ms, mean random = 648 ms; *Z* = −2.1, *P* = 0.03, *N* = 14), while offline, explicit recognition was significantly better than chance (mean = 57.7%, SD = 10%; *Z* = 2.45, *P* = 0.01, *N* = 14; fig. S10) in this cohort.

### Neural tracking of visual SL

We next turned to NFT to identify brain areas exhibiting SL in neurophysiological recordings from 1606 intracranial electrodes in the 12 patients, extensively covering frontal, parietal, temporal, and occipital cortex (fig. S2). To this end, we evaluated within-electrode phase coherence in both the FP and the envelope of the HGB (*15*) in the structured and random streams, respectively. We expected an entrainment response at a 2.66-Hz frequency to individual fractals and at a 1.33-Hz frequency to the learned pairs, the latter only for the structured stream. Providing evidence for the acquisition of regularities, we observed a significant peak in the phase coherence spectrum at the pair rate (i.e., 1.33 Hz), but only for the structured stream (criterion for significance: *P* < 0.05, FDR-corrected; Fig. 3B), and which localized to more posterior regions, predominately occipital and parietal cortex (fig. S12). A significant peak at the fractal rate (i.e., 2.66 Hz) was found for both structured and random streams (criterion for significance: *P* < 0.05, FDR-corrected).

Increases in pair-rate responses were observed, on average, within 430 pair exposures (fig. S13A), with the earliest responses observed in some electrodes by 30 pairs (fig. 13B). Such an increase in the pair-rate response was absent in the random stream ruling out spurious entrainment as a function of time (fig. S13A). Coherence at the pair rate was replicated across subjects, with 12 of 12 patients exhibiting entrainment (table S1).

As in the auditory SL, we observed an anatomical and hierarchical segregation between two temporal tuning profiles of electrodes: one showing significant entrainment at the fractal and pair rates (pair + fractal; Fig. 3D, bottom) and clustered mostly within occipital (striate and extrastriate) and parietal cortex (intraparietal sulcus), and the other showing significant entrainment at the pair-rate only (pair-only; Fig. 3D, top) localized more anteriorly in frontal (middle and superior), parietal, and temporal cortex.

### Representational analysis in visual SL

In the auditory modality, we observed representational changes indicative of sequence learning based on TPs, ordinal position, and word identity across sets of electrodes. How do visual regularities shape neural representations? As learned units in the visual modality contained only two elements (i.e., pairs), grouping based on TPs and ordinal position yields similar results—both cues predict grouping of the first fractal in each pair with the first fractals of other pairs and grouping of the second fractal with the other second fractals. However, although both TP and ordinal position depend on grouping of first fractals together and second fractals together, the representational impact of these cues can be quantified in different, nonexclusive ways (see below). Thus, in the following, we refer to this grouping as consistent with either TP or ordinal position, in contrast with pair identity, which predicts that the first and second members of each pair will be grouped together and different from the other pairs (*9*). Follow-up quantification will allow to differentiate grouping based on TP, ordinal position, and identity.

**Fig. 4:**
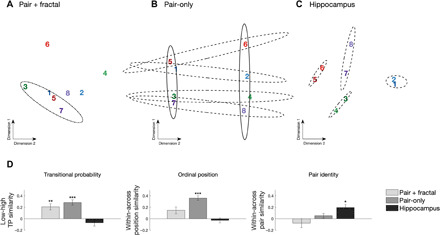
Pattern similarity results during visual SL. MDS of the distances between responses to individual fractals across (**A**) pair-only, (**B**) pair + fractal, and (**C**) hippocampal electrodes. Pairs are color-coded; odd numbers refer to the first position, and even numbers refer to the second position. Dot-dashed ellipses outline grouping by TP/ordinal position in pair + fractal electrodes. Solid ellipses outline grouping by TP/ordinal position in pair-only electrodes. Dashed ellipses indicate grouping by pair in pair-only and hippocampal electrodes. (**D**) Comparison of multivariate pattern similarity for fractals in the visual SL task. Left: Within versus between similarity for low versus high TP. Greater within-class similarity indicates stronger grouping of fractals with a low TP (0.33) over fractals with a high TP (1.0). A Friedman test indicated a main effect of electrode type on TP similarity (χ^2^ = 19.3, *P* < 0.001). Middle: Within versus between similarity for ordinal position. Greater within-class similarity indicates grouping of fractals holding the same first or second position in a pair. A Friedman test indicated a main effect of electrode type (χ^2^ = 122.2, *P* < 0.001). Right: Within versus between similarity for pair identity. Greater within-class similarity indicates grouping of fractals into pairs. A Friedman test indicated a main effect of electrode type (χ^2^ = 40.04, *P* < 0.001). ***P < 001 and *P < 0.05, Wilcoxon rank sum test; error bars denote the population SEM.

We conducted multivariate pattern analysis on the raw FP separately for the sets of electrodes showing pair + fractal responses and pair-only responses, identified through the NFT, in addition to electrodes in the hippocampus. We calculated the correlation distance between the spatial patterns of neural activity across electrodes for every pair of fractals for each of the three electrode sets. We again found that the three sets of electrodes encoded different information. For the pair + fractal electrodes, MDS of the distances between fractals revealed a representation consistent with TPs (representation of first versus second; Fig. 4A). Replicating the auditory findings, for pair-only electrodes, we found concurrent grouping for TPs and/or ordinal position (first dimension) and grouping for pair identity (second dimension) (Fig. 4B). In the hippocampus, however, grouping was only by pair identity (Fig. 4C). Clustering was not observed in any electrode set for the random stream (fig. S16). However, in contrast to the auditory domain, evidence for fast learning during exposure emerged in the first block (fig. S14). These results indicate rapid changes in representational space as a function of visual SL.

To statistically evaluate the groupings, we collapsed pattern similarity across fractals belonging to different classes for each electrode set (pair + fractal, pair-only, and hippocampus). This allowed us to quantify the representational impact of each coding scheme. TP was examined by comparing pattern similarity among first fractals (first-first) with low TP (i.e., relatively unpredictable given preceding fractal) versus among second fractals (second-second) with high transition probability (i.e., predictable given preceding fractal). Ordinal position was examined by comparing pattern similarity within the same position (first-first and second-second) versus between different positions (first-second). Pair identity was examined by comparing pattern similarity within pair (e.g., first_1_-second_1_) versus between pair (first_1_-second_2_).

In line with the MDS, we observed complementary coding across the three sets of electrodes. Pair + fractal electrodes showed greater similarity for low TP, but not for ordinal position or identity. In contrast, pair-only electrodes showed greater similarity for fractals with low TP (Fig. 4D, left) and reliable coding for ordinal position (Fig. 4D, middle) but, in contrast to the auditory modality, not for pair identity (Fig. 4D, right). Last, hippocampal electrodes exclusively showed coding for pair identity. We further analyzed electrode types by field type (e.g., FP-responsive versus HGB-responsive electrodes) and found these general coding strategies preserved by type (word + syll and word-only) and, as in the auditory domain, with the exception that only HGB-responsive electrodes appeared to provide evidence for coding by word identity (fig. S15).

## DISCUSSION

Using intracranial recordings in humans, we have described how the brain tracks and learns structure within sensory information. SL is accompanied by rapid changes in neural representations, reflected in two functionally and anatomically distinct responses: brain regions tracking lower-level sensory input (i.e., syllables and fractals) and higher-order units (i.e., words and pairs) and brain regions only representing learned higher-order units (i.e., words and pairs). These distinct responses reveal an anatomical hierarchy: The former maps onto early, sensory processing stages (e.g., STG and occipital cortex), while the latter encompasses late, amodal processing stages (e.g., IFG and ATL). In other words, while early processing is domain-specific, late processing is domain-general. These nested structures within sensory streams are extracted and represented in the brain in as little as ~2 min, consistent with previous behavioral studies (*2*), and even when subjects are not aware of the process.

Our results are consistent with previous work demonstrating how the cortical hierarchy integrates information over increasingly longer temporal windows (*26*, *27*). Yet, they go beyond topographical mapping of temporal receptive fields. First, we show how SL shapes the neural representational space within these areas profoundly and rapidly. This contrasts with the much more gradual representational change that occurs over development or with longer-term perceptual learning. Second, we discovered that qualitatively different aspects of sequence knowledge are encoded across different brain areas: sites representing the sensory input and higher-order units encode local and generic aspects of sequences, such as their TPs (or degree of uncertainty). In contrast, sites exclusively representing higher-order structure encode more specific aspects of the sequences such as the ordinal position of the elements, but, most importantly, the specific identity of the learned unit. This does not necessarily imply a hierarchy in terms of coding strategies, but rather their coexistence in different brain networks (*12*). The observed representations were only observed in the raw FP data and not when the same analysis was performed on the HGB envelope (see figs. S8 and S17). This specifically points to low-frequency information as a possible mechanism for encoding these representations, frequencies well documented to facilitate information transfer across the cortex and subcortical regions (*28*).

One interesting question for future research is to better understand what is captured by NFT. Previous studies have proposed that frequency results might index perceptual binding of the learned units (*17*). While this mechanism could be at play for the results in the auditory modality, it is unclear how perceptual binding could work for sequentially presented visual stimuli. An alternative possibility, previously discussed, is that NFT might be indexing the activation of areas with different temporal integration windows (*26*). In this account, perceptual changes attributed to perceptual binding could be the consequence from the underlying neural integration. Another alternative is that NFT tracks the increased similarity in the representations as a function of learning, leading to “event boundaries” at times in which dissimilatory is higher (*7*). Unfortunately, given the sparse cortical sampling of ECoG and varying electrode coverages across patients, at present, we cannot dissect these different possibilities.

Previous studies on SL suggest that an increase in predictive uncertainty serves as the primary cue for event segmentation. Our results extend this body of work demonstrating that SL also involves the acquisition of higher-order sequence knowledge, i.e., ordinal position and identity. Higher-order structure or “chunks” may serve as the mental units for mapping segmented word forms onto novel word referents (*22*). These results are in line with the hypothesis that the output of the word boundary discovery may provide cues to edges of constituents, which, in turn, can serve as scaffolding for the subsequent discovery of internal structure, i.e., which elements are contained and in which positions (*29*).

Our finding that sequences are represented at multiple levels, from simple and generic to complex and specific regularities, may reconcile two opposing theoretical models in SL. The “statistical model” posits that learners represent statistical relations between elements in the input and do not explicitly represent statistically coherent units in memory. In contrast, “chunking models” posit that learners represent statistically coherent units of information from the input in memory such that the stored representations are discrete chunks of information. So far, these models have only been contrasted at the behavioral level, e.g., by studying sensitivities to illusory or embedded units (*30*, *31*). An intriguing possibility to be tested in future studies is that these two models actually coexist and map onto the different networks and sequence representations that we report here, i.e., simple representations encoding TPs and complex representations encoding positional information and unit identity. Previously reported discrepancies in behavioral results may reflect the differential engagement of these two neural processes across different tasks. Alternative mechanisms for SL can also be tested using the RSA approach. In particular, two theories can be tested. One posits that SL reflects changes in the similarity space and that transitions are then learned as trajectories through that space (*32*). Another account conceives SL as acquiring a community structure in a symmetric graph with uniform TPs, which are captured by changes in representational similarity (*7*).

The main organizational principles of neural changes underlying SL are shared across the auditory and visual domain. We observed a similar functional clustering of responses, i.e., sensory input + higher-order units and higher-order units only, in both the auditory and visual modalities (compare Figs. 1D with 3D). In addition, no clear hemispheric lateralization was observed for the auditory or visual SL in any of the electrodes following higher-order units, perhaps reflecting the abstract quality of the stimuli. Nevertheless, there were also notable differences between domains: For instance, during the temporal evolution of SL, responses to higher-order units reached group-level significance faster in the visual domain, and with individual electrodes exhibiting significant coherence within 30 pairs versus 50 word exposures (see figs. S5 and S13). This may reflect an artificial difference in the chunk sizes (pair versus triplet) between the two modalities or an innate difference in the learning curves between the auditory and visual learning pathways. In addition, the cortical areas involved in auditory and visual SL only partially overlapped (fig. S18). This was to be expected at the level of the sensory responses: Responses encoding the sensory input and higher-order units clustered around the STG for the auditory SL involving syllables and around occipital cortex for the visual SL involving fractals. Perhaps more interestingly, areas engaged exclusively in higher-order unit representation were also partially separated. While areas such as IFG and ATL tracked higher-order units in both modalities, middle frontal and superior parietal cortices seemed to be differentially involved in auditory and visual SL, respectively. This result cannot be explained by the fact that different groups of subjects (and electrode coverage) contributed to the different tasks, as we confirmed this functional separation in six subjects who completed both auditory and visual SL tasks (fig. S18).

This suggests that while sequence operations performed across domains might build on similar representations (TP, ordinal position, and identity), the circuits performing these operations might be modality-specific to some extent, much like the nested tree structures involved in language, music, and mathematics that are each represented in distinct circuits. Our results speak for a more modularized representation of sequences for the encoding of local and simple aspects such as TP represented in sensory areas but a less modularized representation as complexity increases, as IFG and ATL encoded positional and identity information for both visual and auditory SL, in line with a domain-general role in SL. In turn, the hippocampus, at the top of the hierarchy, uniquely represents the identity of both visual and auditory sequences (*33*).

To our knowledge, the complementary representation of sequences across the cortex and the hippocampus, with a gradient of abstraction, has not been previously reported for SL. Our findings shed light on the elementary operations during SL and how cortex and hippocampus differentially support these processes. For instance, lower-level cortical coding based on TPs could facilitate initial segmentation, as uncertainty drives prediction errors and boundaries. Coding based on TPs, while a powerful cue to discover boundaries in the continuous stream, does not easily accommodate the integration and binding across elements. Higher-order cortical encoding based on ordinal position permits novel recombination of elements to create unique entities. In this case, as long as ordinal position is respected, novel recombination of elements can be allowed. Last, hippocampal integration and binding across stable combinations of units facilitate the attachment of meaning or identity. Thus, a great benefit of the observed complementary coding across the cortex and the hippocampus is that it may allow the further use of those information for different cognitive operations. These complementary roles of the cortex and the hippocampus were observed even during a brief exposure (<10 min). An interesting question for future research is to investigate the stability of these functions across longer exposure, and whether complementary coding persists or is replaced for a winner-take-all coding depending in the number of repetitions. Another question relates to how sleep consolidation affects these complementary representations.

An unexpected observation was the dissociation between “online” and “offline” behavioral measures of SL. Through frequency tagging, we were able to localize with precision the areas involved in the acquisition of higher-order regularities in the structured stream—a response found in virtually all participants and that increased as a function of exposure. Furthermore, representational analysis demonstrated learning of TP, ordinal position, and unit identity after short exposure. Faster reaction times during the cover task showed that structured stream presentation facilitated learning behavior. Yet, the patients did not perform better than chance in the subsequent “offline” behavioral recognition test. In neurotypical subjects, however, we observed facilitation in the reaction time in the structured stream and subsequent above-chance recognition performance in the offline behavioral task. In other words, cortical circuits for automatic SL appear intact in our patients, while episodic memory appears impaired. Episodic memory dysfunction is prevalent in temporal lobe epilepsy (*34*). One aspect worth considering relates to the validity of the indirect, online tasks to measure learning, because they have been recently called into question (*35*). Changes in reaction time (RT) have been attributed to confounds, i.e., serial order in short sequences with clear onsets and/or anchoring cues. While this confound might play a role in certain tasks, it is unlikely to explain our results for several reasons: In our materials, the onset and offset of each sequence was ramped to prevent a clear anchoring effect effectively removing a cue for serial order; also, subjects performed the RT task during the 2-min-long sequence and not just over a couple of exemplar token as done in other studies. Furthermore, repetitions were inserted in both the random and structured stream, preserving the order in the long list. With all those controls at hand, we believe our results index learning in both the patient and neurotypical population, making the dissociation all the more relevant when considering that NFT correlated only with learning in the online and not in the offline task.

Relatedly, the fact that we observed SL in patients with epilepsy may challenge the importance of the medial temporal lobe and hippocampus for SL. Previous functional magnetic resonance imaging (fMRI) studies in healthy subjects have demonstrated that visual SL leads to changes in representational similarity in the hippocampus (*9*), which we also observed in our population of patients. The critical role of the hippocampus has been corroborated by lesion studies in humans in visual and auditory SL (*10*, *23*). Computational models indicate a division of labor in the hippocampus between the monosynaptic pathway (connecting entorhinal cortex directly to CA1), which supports SL, and the trisynaptic pathway (connecting entorhinal cortex to CA1 through dentate gyrus and CA3), which supports episodic memory (*36*). Given that epilepsy can lead to selective deficits in hippocampal circuits (*37*), the observed dissociation between online and offline behavioral measures of SL could reflect disproportionate damage to the trisynaptic pathway.

The dissociation between online and offline behavioral measures is also noteworthy given the large variability observed when SL is measured through explicit behavioral tasks (*38*). NFT may provide a more sensitive and robust measure of learning compared to explicit tasks, even in healthy populations. One limitation of this technique, and intracranial recordings in general, is the inability to perform meaningful comparisons across subjects (e.g., brain-behavior correlation) to investigate whether the strength of NFT is directly related to behavioral outcomes (i.e., explicit recognition). While we did observe a significant relationship between the strength of neural entrainment and online reaction times, we did not observe any relationship with offline recognition (figs. S3 and S11). Because of differences in electrode coverage across patients, it was not possible to determine whether the lack of brain-behavior correlation with the offline measure of learning is due solely to disparate sampling of the cortex, and we therefore urge caution when interpreting these results. However, this technique opens up exciting opportunities to characterize learning trajectories across clinical and healthy populations, across sensory modalities. Because NFT does require task demands, it is well suited to tracking the acquisition of sequence knowledge across the life span from newborns to the elderly and even in cognitively impaired patients. The combination of NFT with RSA provides a powerful toolkit to reveal how the brain engages in SL rapidly across multiple levels of organization in the human brain.

## MATERIALS AND METHODS

### Stimulus materials and summary of experimental procedures

#### Auditory SL task

Twelve consonant-vowel syllables were synthetically generated using MacTalk. Syllable lengths were equated, and prosody was flattened using Praat (*39*). The individual syllables were concatenated in MATLAB. Two sequences were created: a structured and a random sequence. In the structured sequence, TPs between syllables were manipulated such that four hidden words (three syllables each) were embedded in the sequence (see Fig. 1), resulting in a continuous artificial language stream with an underlying syllable presentation rate of 4 Hz and word rate of 1.33 Hz. In the random sequence, TPs across syllables were the same (e.g., *P* = 1/11 syllables). Each sequence lasted approximately 2 min (540 syllable presentations) and was presented five times. To avoid potential cueing of the words at the start and end of the stream, the volume of the audio stream was ramped on and off, over the first and last 1.5 s, respectively. Participants were not informed of the structure, and instead, to ensure task compliance, participants were asked to perform a cover task in which they indicated syllable repetitions that were randomly embedded in the auditory streams. Sixteen syllable repetitions were randomly embedded into each presentation block of a sequence.

Once both streams (random and structured) had been played to the participants, they were then informed that one of the audio streams consisted of a hidden structure containing four “words.” A “word” was defined to the participants as a three-syllable nonsense word that was repeated in the stream. Subjects then performed a 2AFC task where they listened to two audio segments, presented one after the other, and asked to select the one containing one of the hidden words. One audio segment contained one of the words (e.g., “tupiro”) that was embedded in the structured sequence, while the other segment was a lower probability “part-word” from the structured stream that spanned word boundaries (e.g., “labutu” composed of golabu + tupiro). Sixteen trials, consisting of all possible word versus part-word combinations, were presented. Presentation order of the word in the first and second audio segments was counterbalanced across trials. Because exposure to the individual syllables is equated, a preference for the true words over the part-word is indicative of SL.

#### Visual SL task

The procedure for the visual SL task was identical to the auditory SL task; however, in this task, sequences were formed from eight fractals (four sets of two fractal pairs, duplets). Fractals were taken from the same set of images previously used in (*9*). The stimulus-onset asynchrony between fractals was set to 375, whereby each fractal was presented for 233 ms with an interstimulus interval of 150 ms. In the structured sequence, TPs between fractals were manipulated such that four hidden fractal pairs (two fractal each) were embedded in the sequence (see Fig. 3), resulting in a continuous stream of fractals with a presentation rate of 2.6 Hz and a fractal-pair rate of 1.3 Hz. In the random sequence, TPs remained fixed between all possible fractals (e.g., *P* = 1/7). Each sequence lasted approximately 2 min (360 fractal presentations). As in the auditory learning task, participants were not informed of the structure; however, to ensure task compliance, participants were asked to perform a cover task in which they indicated, using the keyboard, when a fractal had been repeated. Sixteen fractal repetitions were randomly embedded within each sequence block. After exposing participants to both streams (random and structured), the experimenter disclosed to them that one stream contained four pairs of fractals forming a duplet that repeated across the stream. Participants then completed 16 two-alternative forced-choice test trials, in which they judge the relative familiarity of a duplet from the exposure phase compared to a foil composed of a pair of fractals that spanned the pair boundary (e.g., fractals 1 to 2 versus 4 to 6).

### Participants and recordings

#### Electrocorticography

ECoG recordings were obtained from a total of 23 patients (13 female, average age of 35 years, range of 16 to 59 years, 21 right-handed) with drug-resistant focal epilepsy undergoing clinically motivated invasive monitoring at the Comprehensive Epilepsy Center of the New York University Langone Medical Center. Eleven subjects participated in the auditory SL only, 6 subjects in the visual SL only, and 6 subjects participating in both the auditory and visual SL task. All subjects participating in the study provided oral and written informed consent before participation in the study, in accordance with the Institutional Review Board (IRB) at the New York University Langone Medical Center. Patients were informed that participation in the study would not affect their clinical care and that they could withdraw from the study at any point without affecting medical treatment. Brain activity was recorded from a total of 3689 (average of 120 ± 30 per subject) intracranially implanted subdural platinum-iridium electrodes embedded in silastic sheets (2.3-mm-diameter contacts, Ad-Tech Medical Instrument). The decision to implant, electrode targeting, and the duration of invasive monitoring were determined solely on clinical grounds without reference to this or any other study. Macroelectrodes were arranged as grid arrays (8 × 8 contacts, 10- or 5-mm center-to-center spacing), linear strips (1 × 8/12 contacts), or depth electrodes (1 × 8/12 contacts), or some combination thereof. Subdural electrodes covered extensive portions of lateral and medial frontal, parietal, occipital, and temporal cortex of the left and/or right hemisphere (see fig. S1 for electrode coverage across all subjects and for the individual coverage of each subject). Recordings from grid, strip, and depth electrode arrays were acquired using a NicoletOne C64 clinical amplifier (Natus Neurologics, Middleton, WI), bandpass-filtered from 0.16 to 250 Hz, and digitized at 512 Hz. Intracranial EEG signals were referenced to a two-contact subdural strip facing toward the skull near the craniotomy site. Data were subsequently downsampled to 250 Hz, and a 60-Hz notch filter was applied to remove any line-noise artifacts. All electrodes were visually inspected; those with excessive noise artifacts were removed from subsequent analysis (185 of 3689 electrodes removed).

### Data analysis

#### ECoG surface reconstruction and electrode localization

Presurgical and postsurgical T1-weighted MRIs were acquired for each patient, and the location of the electrode relative to the cortical surface was determined from co-registered MRIs following the procedure described by Yang and colleagues (*40*). Co-registered, skull-stripped T1 images were nonlinearly registered to an MNI-152 template, and electrode locations were then extracted in Montreal Neurological Institute (MNI) space (projected to the surface) using the co-registered image. A three-dimensional reconstruction of each patient’s brain was computed using FreeSurfer (http://surfer.nmr.mgh.harvard.edu). In all figures, electrode locations are projected onto the left hemisphere of the MNI-152 template brain, unless otherwise noted.

### Behavioral data analysis

Performance during the online incidental task was assessed by calculating a *d*′ score for every participant across all five exposure trials. As the incidental task was embedded in the continuous stream of auditory or visual stimuli, detection of a syllable or image repetition was deemed accurate (“hit”) if the participant made a keyboard response within 250 to 1500 ms of the occurrence of the repetition (results are robust to the selection of the response window, as comparable results were obtained using response windows up to 750, 1000, and 3000 ms). All other keyboard responses outside the valid response window were deemed false alarms. Significance of *d*′ scores were assessed via a one-sample *t* test, and comparison of *d*′ scores across conditions (structured versus random) was assessed with a paired *t* test. Analysis of reaction times between conditions was assessed using a Wilcoxon two-sided paired signed-rank test between the average reaction times per condition and participant. Four participants who participated in the visual SL task were excluded from this analysis: three for excessive button pressing, and one who did not perform this task because they were confused about the task directives. Performance on the offline 2AFC explicit recognition test was assessed by determining the percentage of correctly identified “words” or “fractal pairs” and subjected to a Wilcoxon signed-rank test against chance performance (50%). Two of the 17 patients who participated in the auditory SL experiment did not complete the 2AFC task: one for technical reasons and one because the participant was confused about the task. One of the 12 patients who participated in the visual SL experiment did not complete the 2AFC task because the participant was confused about the task.

### NFT (phase coherence analysis)

The main hypothesis of this study was that SL could identified by tagging electrodes that were entrained at the fundamental unit frequency [e.g., word rate and pair rate; see (*15*, *41*)]. Therefore, we used phase coherence analysis as a means to identify (“tag”) electrodes that exhibited significant phase coherence at the fundamental unit frequency at 1.33 Hz. For each experiment, the raw signals (FPs) from all five blocks of a sequence (structured or random) were concatenated and then reshaped into 10-word segments (10 words × 90 trials × electrodes) and converted into the frequency domain via fast Fourier transform (0.134-Hz resolution). Phase coherence was computed for each electrode, $R^{2}={\left[\sum^{N}\text{cos}\emptyset \right]}^{2}+{\left[\sum^{N}\text{sin}\emptyset \right]}^{2}$, over the 90 trials (*42*). Significance of the response at each frequency of interest (e.g., 1.33 and 4 Hz) was determined by comparing the magnitude of the coherence response to 1000 phase-shuffled surrogate datasets and then subjected to false discovery rate (FDR) correction across all electrodes per subject using the Benjamini-Hochberg procedure (*43*). Using this technique, we identified FP responsive in all electrodes across all subjects that exhibited a significant peak at 1.33 Hz (criterion for electrode selection, *P* < 0.05 after FDR correction). From this set of electrodes, we identified two subsets of responses: electrodes that exhibited a significant peak at the unit + stimulus (e.g., word + syll and pair + fractal) and electrodes that only exhibited a significant peak in phase coherence spectrum at the fundamental unit rate (e.g., word-only and pair-only). The same analysis was performed on the envelope of the HGB signals, extracted using eight semi-logarithmically spaced constant-Q Morlet wavelet filters (center frequencies between 70 and 150 Hz), subsequently averaged across frequency bands and taking the absolute value of the Hilbert transform of the averaged response, identifying a set of HGB-responsive electrodes for further analysis. Because ECoG recordings cannot be averaged across subjects, we pooled all identified FP- and HGB-responsive electrodes across subjects. We later control for the effects of individual electrodes using a random resampling technique (see the “Within versus between category similarity analysis” section).

### Localization and region of interest analysis

Electrodes identified by the NFT as responsive were plotted on a standard brain using the MNI coordinates. The number of responsive electrodes per subject and per field type (FP and HGB) was analyzed per region of interest (ROI) based on the Desikan-Killiany cortical atlas (figs. S4 and S12) (*44*). To better understand which regions of the brain showed these word-rate and pair-rate responses, we grouped electrodes in six major ROIs and analyzed the proportion of electrodes that were found to be responsive in a given ROI. We then compared these proportions across ROIs and calculated the odds ratio of a given ROI contributing more responsive electrodes than another (figs. S4 and S12). This provides an estimate of which brain regions are more or less active during the task.

### Phase coherence latency analysis

The response latency was computed for all responsive electrodes with a significant phase coherence at the higher-order rate (i.e., word rate or pair rate, 1.33 Hz) determined across all blocks. To identify a time point when each electrode exhibited an initial significant response (e.g., time to first significant response), phase coherence was computed by sequentially adding in trials (10 words per trial) and evaluating the phase consistency at 1.33 Hz using a Rayleigh test (*45*). The time point (in number of words, e.g., trials) at which each electrode reached significance (FDR-corrected across all tested electrodes) was estimated for each electrode (see figs. S5B and S13B). In addition, *P* values were averaged across all responsive electrodes and compared across task conditions (structured versus random) to access differences in phase coherence across conditions (figs. S5A and S13A).

### Representational similarity analysis

To assess the similarity of neural responses to each token (syllables and fractals), a multivariate spatial pattern analysis was performed on all responsive electrodes (FP-responsive + HGB-responsive). First, all individual trials were “whitened” by the noise covariance matrix computed across all tokens and trials (*46*), and the average response across all tokens (e.g., nonspecific response) was subtracted from each trial. Next, the data were vectorized across all responsive electrodes (e.g., samples × significant electrodes) within an electrode subtype (e.g., word + syll or word-only), and the dissimilarity of the spatial pattern vectors was computed between each pair of tokens. Dissimilarity was assessed using cross-validated correlation distance between tokens (e.g., syllables “tu” versus “pi”), and using fivefold cross-validation. All neural responses to tokens (e.g., trials) were split into five folds, using 20% of the data in a fold as the validation set. For each fold, a cross-validated correlation distance was computed, where the pattern vectors of one partition (the training set) are projected onto the pattern vectors of an independent dataset validation set (*46*). The resultant representational dissimilarity matrix was subjected to a principal components analysis, and the first two dimensions were plotted to produce a two-dimensional visualization of dissimilarity scores across all pairs (see Fig. 2). Subsequently, quantification of the representational spaces (i.e., similarity of neural responses between tokens) was estimated by comparing within versus between category similarity using hypothesized models of feature encoding (see the next section).

### Within versus between category similarity analysis

To quantify the degree to which responses are able to capture features of the learned streams, i.e., TP, ordinal position, and/or identity, we calculated the difference in similarity (Pearson correlation) between items in the same category and items belonging to the other category in question. To control for the effects of any one electrode driving the similarity, this estimate was calculated by randomly sampling the responsive electrodes by type (e.g., FP and word-only) and computing a similarity matrix between all tokens (e.g., syllables or fractals), for each resampling. This resampling procedure was repeated 200 times (with replacement), and the average Fisher-transformed correlations of all elements within the same category (within category) were compared against all items that spanned an opposing category (between category) using a Wilcoxon rank sum test (two-sided). For example, in assessing ordinal position coding with three tokens per word (i.e., auditory task), the similarity between token pairs within the same category (e.g., all first position comparisons, all second position comparisons, and all third position comparisons) were compared to the similarity of token pairs that crossed between this category (first position versus second position, first position versus third position, etc.). To evaluate TP coding, all token pairs with the same low TP (all first position comparisons, within category) were compared against all token pairs with high TP (all second position comparisons + all second position − third position comparisons, between category). For identity coding, the similarity between token pairs within the same word (within category) was compared to the similarity between all tokens that spanned different words (between category), excluding all ordinal position comparisons (all first position comparisons, all second position comparisons, etc.). See fig. S19 for a visualization of the within-category versus between-category comparisons.

## SUPPLEMENTARY MATERIALS

Supplementary material for this article is available at http://advances.sciencemag.org/cgi/content/full/7/8/eabc4530/DC1

## Appendix

View/request a protocol for this paper from *Bio-protocol*.

## References

[1] P. K. Kuhl, Early language acquisition: Cracking the speech code. Nat. Rev. Neurosci. 5, 831–843 (2004). 15496861 10.1038/nrn1533

[2] J. R. Saffran, R. N. Aslin, E. L. Newport, Statistical learning by 8-month-old infants. Science 274, 1926–1928 (1996). 8943209 10.1126/science.274.5294.1926

[3] N. Z. Kirkham, J. A. Slemmer, S. P. Johnson, Visual statistical learning in infancy: Evidence for a domain general learning mechanism. Cognition 83, B35–B42 (2002). 11869728 10.1016/s0010-0277(02)00004-5

[4] N. B. Turk-Browne, in *The Influence of Attention, Learning, and Motivation on Visual Search*, M. D. Dodd, J. H. Flowers, Eds. (Springer, 2012), pp. 117–146. 23437627

[5] R. N. Aslin, Statistical learning: A powerful mechanism that operates by mere exposure. Wiley Interdiscip. Rev. Cogn. Sci. 8, e1373 (2017). 10.1002/wcs.1373 PMC5182173 27906526

[6] G. Orbán, J. Fiser, R. N. Aslin, M. Lengyel, Bayesian learning of visual chunks by human observers. Proc. Natl. Acad. Sci. U.S.A. 105, 2745–2750 (2008). 18268353 10.1073/pnas.0708424105 PMC2268207

[7] A. C. Schapiro, T. T. Rogers, N. I. Cordova, N. B. Turk-Browne, M. M. Botvinick, Neural representations of events arise from temporal community structure. Nat. Neurosci. 16, 486–492 (2013). 23416451 10.1038/nn.3331 PMC3749823

[8] M. Maheu, S. Dehaene, F. Meyniel, Brain signatures of a multiscale process of sequence learning in humans. eLife 8, e41541 (2019). 30714904 10.7554/eLife.41541 PMC6361584

[9] A. C. Schapiro, L. V. Kustner, N. B. Turk-Browne, Shaping of object representations in the human medial temporal lobe based on temporal regularities. Curr. Biol. 22, 1622–1627 (2012). 22885059 10.1016/j.cub.2012.06.056 PMC3443305

[10] A. C. Schapiro, E. Gregory, B. Landau, M. McCloskey, N. B. Turk-Browne, The necessity of the medial temporal lobe for statistical learning. J. Cogn. Neurosci. 26, 1736–1747 (2014). 24456393 10.1162/jocn_a_00578 PMC4264662

[11] K. McNealy, J. C. Mazziotta, M. Dapretto, Cracking the language code: Neural mechanisms underlying speech parsing. J. Neurosci. 26, 7629–7639 (2006). 16855090 10.1523/JNEUROSCI.5501-05.2006 PMC3713232

[12] S. Dehaene, F. Meyniel, C. Wacongne, L. Wang, C. Pallier, The neural representation of sequences: From transition probabilities to algebraic patterns and linguistic trees. Neuron 88, 2–19 (2015). 26447569 10.1016/j.neuron.2015.09.019

[13] R. Frost, B. C. Armstrong, N. Siegelman, M. H. Christiansen, Domain generality versus modality specificity: The paradox of statistical learning. Trends Cogn. Sci. 19, 117–125 (2015). 25631249 10.1016/j.tics.2014.12.010 PMC4348214

[14] M. Buiatti, M. Peña, G. Dehaene-Lambertz, Investigating the neural correlates of continuous speech computation with frequency-tagged neuroelectric responses. Neuroimage 44, 509–519 (2009). 18929668 10.1016/j.neuroimage.2008.09.015

[15] N. Ding, L. Melloni, H. Zhang, X. Tian, D. Poeppel, Cortical tracking of hierarchical linguistic structures in connected speech. Nat. Neurosci. 19, 158–164 (2016). 26642090 10.1038/nn.4186 PMC4809195

[16] N. B. Turk-Browne, J. Jungé, B. J. Scholl, The automaticity of visual statistical learning. J. Exp. Psychol. Gen. 134, 552–564 (2005). 16316291 10.1037/0096-3445.134.4.552

[17] L. J. Batterink, K. A. Paller, Online neural monitoring of statistical learning. Cortex 90, 31–45 (2017). 28324696 10.1016/j.cortex.2017.02.004 PMC5438777

[18] H. Getz, N. Ding, E. L. Newport, D. Poeppel, Cortical entrainment to constituent structure in language acquisition. Cognition 181, 135–140 (2018). 30195135 10.1016/j.cognition.2018.08.019 PMC6201233

[19] J. R. Saffran, E. L. Newport, R. N. Aslin, Word segmentation: The role of distributional cues. J. Mem. Lang. 35, 606–621 (1996).

[20] G. Hickok, D. Poeppel, The cortical organization of speech processing. Nat. Rev. Neurosci. 8, 393–402 (2007). 17431404 10.1038/nrn2113

[21] A. D. Endress, J. Mehler, The surprising power of statistical learning: When fragment knowledge leads to false memories of unheard words. J. Mem. Lang. 60, 351–367 (2009).

[22] K. Graf Estes, J. L. Evans, M. W. Alibali, J. R. Saffran, Can infants map meaning to newly segmented words? Statistical segmentation and word learning. Psychol. Sci. 18, 254–260 (2007). 17444923 10.1111/j.1467-9280.2007.01885.x PMC3864753

[23] N. V. Covington, S. Brown-Schmidt, M. C. Duff, The necessity of the hippocampus for statistical learning. J. Cogn. Neurosci. 30, 680–697 (2018). 29308986 10.1162/jocn_a_01228 PMC5876146

[24] H. Nili, C. Wingfield, A. Walther, L. Su, W. Marslen-Wilson, N. Kriegeskorte, A toolbox for representational similarity analysis. PLOS Comput. Biol. 10, e1003553 (2014). 24743308 10.1371/journal.pcbi.1003553 PMC3990488

[25] J. M. Zacks, B. Tversky, Event structure in perception and conception. Psychol. Bull. 127, 3–21 (2001). 11271755 10.1037/0033-2909.127.1.3

[26] U. Hasson, J. Chen, C. J. Honey, Hierarchical process memory: Memory as an integral component of information processing. Trends Cogn. Sci. 19, 304–313 (2015). 25980649 10.1016/j.tics.2015.04.006 PMC4457571

[27] C. J. Honey, T. Thesen, T. H. Donner, L. J. Silbert, C. E. Carlson, O. Devinsky, W. K. Doyle, N. Rubin, D. J. Heeger, U. Hasson, Slow cortical dynamics and the accumulation of information over long timescales. Neuron 76, 423–434 (2012). 23083743 10.1016/j.neuron.2012.08.011 PMC3517908

[28] L. H. Arnal, A.-L. Giraud, Cortical oscillations and sensory predictions. Trends Cogn. Sci. 16, 390–398 (2012). 22682813 10.1016/j.tics.2012.05.003

[29] A. D. Endress, L. L. Bonatti, Words, rules, and mechanisms of language acquisition. Wiley Interdiscip. Rev. Cogn. Sci. 7, 289 (2016). 26683248 10.1002/wcs.1376

[30] P. Perruchet, B. Poulin-Charronnat, Beyond transitional probability computations: Extracting word-like units when only statistical information is available. J. Mem. Lang. 66, 807–818 (2012).

[31] L. K. Slone, S. P. Johnson, When learning goes beyond statistics: Infants represent visual sequences in terms of chunks. Cognition 178, 92–102 (2018). 29842989 10.1016/j.cognition.2018.05.016 PMC6261783

[32] F. H. Wang, E. A. Hutton, J. D. Zevin, Statistical learning of unfamiliar sounds as trajectories through a perceptual similarity space. Cognit. Sci. 43, e12740 (2019). 31446661 10.1111/cogs.12740

[33] N. B. Turk-Browne, The hippocampus as a visual area organized by space and time: A spatiotemporal similarity hypothesis. Vision Res. 165, 123–130 (2019). 31734633 10.1016/j.visres.2019.10.007 PMC6881556

[34] B. Lemesle, M. Planton, B. Pagès, J. Pariente, Accelerated long-term forgetting and autobiographical memory disorders in temporal lobe epilepsy: One entity or two? Rev. Neurol. 173, 498–505 (2017). 28843413 10.1016/j.neurol.2017.07.004

[35] K. Himberger, A. Finn, C. Honey, Reconsidering the automaticity of visual statistical learning. PsyArXiv. 10.31234/osf.io/r659w.

[36] A. C. Schapiro, N. B. Turk-Browne, M. M. Botvinick, K. A. Norman, Complementary learning systems within the hippocampus: A neural network modelling approach to reconciling episodic memory with statistical learning. Philos. Trans. R. Soc. Lond. B Biol. Sci. 372, 20160049 (2017). 27872368 10.1098/rstb.2016.0049 PMC5124075

[37] F. Zhao, H. Kang, L. You, P. Rastogi, D. Venkatesh, M. Chandra, Neuropsychological deficits in temporal lobe epilepsy: A comprehensive review. Ann. Indian Acad. Neurol. 17, 374–382 (2014). 25506156 10.4103/0972-2327.144003 PMC4251008

[38] N. Siegelman, R. Frost, Statistical learning as an individual ability: Theoretical perspectives and empirical evidence. J. Mem. Lang. 81, 105–120 (2015). 25821343 10.1016/j.jml.2015.02.001 PMC4371530

[39] P. Boersma, D. Weenink, Praat: doing phonetics by computer [Computer program]. Version 6.0.19, (2016). retrieved 13 June 2016 from http://www.praat.org/.

[40] A. I. Yang, X. Wang, W. K. Doyle, E. Halgren, C. Carlson, T. L. Belcher, S. S. Cash, O. Devinsky, T. Thesen, Localization of dense intracranial electrode arrays using magnetic resonance imaging. Neuroimage 63, 157–165 (2012). 22759995 10.1016/j.neuroimage.2012.06.039 PMC4408869

[41] J. Farthouat, A. Franco, A. Mary, J. Delpouve, V. Wens, M. Op de Beeck, X. De Tiège, P. Peigneux, Auditory magnetoencephalographic frequency-tagged responses mirror the ongoing segmentation processes underlying statistical learning. Brain Topogr. 30, 220–232 (2017). 27613530 10.1007/s10548-016-0518-y

[42] N. Ding, J. Z. Simon, Power and phase properties of oscillatory neural responses in the presence of background activity. J. Comput. Neurosci. 34, 337–343 (2013). 23007172 10.1007/s10827-012-0424-6 PMC3543520

[43] Y. Benjamini, D. Yekutieli, The control of the false discovery rate in multiple testing under dependency. Ann. Stat. 29, 1165–1188 (2001).

[44] R. S. Desikan, F. Ségonne, B. Fischl, B. T. Quinn, B. C. Dickerson, D. Blacker, R. L. Buckner, A. M. Dale, R. P. Maguire, B. T. Hyman, M. S. Albert, R. J. Killiany, An automated labeling system for subdividing the human cerebral cortex on MRI scans into gyral based regions of interest. Neuroimage 31, 968–980 (2006). 16530430 10.1016/j.neuroimage.2006.01.021

[45] P. Berens, CircStat: A MATLAB toolbox for circular statistics. J. Stat. Softw. 31, 1–21 (2009).

[46] A. Walther, H. Nili, N. Ejaz, A. Alink, N. Kriegeskorte, J. Diedrichsen, Reliability of dissimilarity measures for multi-voxel pattern analysis. Neuroimage 137, 188–200 (2016). 26707889 10.1016/j.neuroimage.2015.12.012

